# Symptom-agnostic endpoints in tuberculosis vaccine efficacy trials

**DOI:** 10.1016/j.langlo.2026.104079

**Published:** 2026-10-01

**Authors:** Morten Ruhwald, Alemnew F Dagnew, Gavin Churchyard, Adam Penn-Nicholson, Puneet Dewan, Alberto L Garcia-Basteiro, Emily B Wong, Willem A Hanekom, Mark Hatherill, Reinout van Crevel, Saskia den Boon, Patricia Hall-Eidson, Rein M G J Houben, Ann M Ginsberg, Frank Cobelens, Katherine C Horton, Richard G White

**Affiliations:** a Novo Nordisk Foundation Initiative for Vaccines and Immunity, Copenhagen, Denmark; b Gates Medical Research Institute, Clinical Development, Cambridge, MA, USA; c The Aurum Institute, Johannesburg, South Africa; d School of Public Health, University of Witwatersrand, Johannesburg, South Africa; e Department of Medicine, Vanderbilt University Medical Center, Nashville, TN, USA; f Foundation for Innovative New Diagnostics (FIND), Geneva, Switzerland; g Gates Foundation, Seattle, WA, USA; h Barcelona Institute for Global Health, Barcelona, Spain; i Hospital Clínic-Universitat de Barcelona, Barcelona, Spain; j Centro de Investigação em Saúde de Manhiça, Maputo, Mozambique; k Africa Health Research Institute, Durban and Somkhele, KwaZulu-Natal, South Africa; l Division of Infectious Diseases, Heersink School of Medicine, University of Alabama Birmingham, Birmingham, AL, USA; m South African Tuberculosis Vaccine Initiative, Institute of Infectious Disease & Molecular Medicine, Division of Immunology, Department of Pathology, University of Cape Town, Cape Town, South Africa; n Radboud University Medical Center, Nijmegen, Netherlands; o Blizard Institute, Queen Mary University of London, London, UK; p Department of Immunization, Vaccines and Biologicals, WHO, Geneva, Switzerland; q Department for HIV, Tuberculosis, Hepatitis and Sexually Transmitted Infections, WHO, Geneva, Switzerland; r Department of Infectious Disease Epidemiology, Faculty of Epidemiology & Population Health, London School of Hygiene & Tropical Medicine, London, UK; s TB Modelling Group, TB Centre, Vaccine Centre and Centre for Mathematical Modelling of Infectious Diseases, London School of Hygiene & Tropical Medicine, London, UK; t Boulder, CO, USA; u Department of Global Health and Amsterdam Institute for Global Health and Development, Amsterdam University Medical Centers, University of Amsterdam, Amsterdam, Netherlands

## Abstract

Tuberculosis vaccine prevention-of-disease efficacy trials traditionally rely on symptom-triggered case detection. This approach captures only a subset of bacteriologically confirmed disease and can miss cases that occur between scheduled trial visits or outside trial procedures, slowing endpoint accrual and complicating interpretation of results. At the same time, the diagnostic landscape for tuberculosis is changing rapidly. Advances, including near point-of-care (NPOC) molecular assays using sputum and non-sputum specimens, portable chest radiography with computer-aided detection, and digital tools for decentralised monitoring, now make symptom-agnostic screening strategies increasingly feasible in large clinical trials. A 2026 WHO policy recommending the use of NPOC molecular diagnostics for tuberculosis further highlights the relevance of these approaches. In this Health Policy paper, we outlined a framework for integrating symptom-agnostic screening into tuberculosis vaccine efficacy trials and reviewed emerging diagnostic technologies that could enable this transition. Using parameters informed by the placebo arm of the M72/AS01_E_ phase 2b trial, we present a worked example illustrating a possible effect on endpoint detection and case-verification burden. In this illustrative scenario, an NPOC-based symptom-agnostic screening strategy was estimated to increase detection of bacteriologically confirmed tuberculosis endpoints by approximately 1·4-fold, as compared with that by symptom-based screening alone, although this finding should not be interpreted as a direct estimate of overall trial efficiency. Realising these benefits will require analytical validation of diagnostic tools, clinical qualification for trial use, and early alignment with regulators and policy bodies. If evaluated through staged evidence generation and implemented with stringent confirmation algorithms, then symptom-agnostic screening could support more interpretable assessments of vaccine impact across the bacteriological spectrum of tuberculosis disease while preserving endpoint specificity.

## Reframing future tuberculosis vaccine efficacy trials in the era of next-generation diagnostics

Tuberculosis remains the leading cause of infectious disease mortality due to a single pathogen, and effective vaccines are a global priority.^1^ However, clinical evaluation of tuberculosis vaccines poses unique challenges.^2^ Traditionally, tuberculosis vaccine prevention-of-disease (POD) efficacy trials have relied on symptom-triggered microbiological confirmation, with participants followed-up for several years and contacted every few months for symptom screening in addition to the scheduled trial site visits.^3^, ^4^, ^5^, ^6^ Those reporting clinical signs or symptoms of pulmonary tuberculosis at screening undergo case investigation, which typically consists of three visits within a 7-day period, during which three sputum samples are collected for culture and molecular assay testing such as with Xpert MTB/RIF or Xpert MTB/RIF Ultra.^5^, ^6^, ^7^ The M72/AS01_E_ phase 2b trial defined a laboratory-confirmed pulmonary tuberculosis case as having at least one of up to six test results positive,^8^ whereas the ongoing M72/AS01_E_ phase 3 and MTBVAC phase 2b trials in adolescents and adults both define a laboratory-confirmed pulmonary tuberculosis case as having at least two of up to six test results positive, obtained from the same or different sputum samples before initiation of tuberculosis treatment.^3^^,^^5^^,^^6^

This design implicitly defines the spectrum of tuberculosis disease that can be detected as trial endpoints: only symptomatic clinical tuberculosis is detected, whereas the populations with asymptomatic disease are not ascertained at all.^3^^,^^4^^,^^9^, ^10^, ^11^ Endpoint accrual is slower when only symptom screen-positive bacteriologically confirmed tuberculosis is detected, as compared with that in approaches that detect all cases of bacteriologically confirmed tuberculosis irrespective of symptom screening.^9^^,^^12^

The M72/AS01_E_ clinical programme also illustrates the operational complexity of endpoint capture in tuberculosis vaccine trials. In the placebo arm of the phase 2b trial, only 26 of the 33 sputum Xpert MTB/RIF-confirmed or culture-confirmed (or confirmed by both), pulmonary tuberculosis cases identified during the study period were included in the primary endpoint analysis (78·8% [95% CI 61·1–91·0]). The remaining seven cases had had symptoms, sought tuberculosis care outside of the trial, and initiated tuberculosis treatment before presenting to the trial site. These cases were, therefore, excluded from the primary per protocol analysis^8^ (third case definition, n=6), with one additional case excluded because the sputum sample was collected 54 days after treatment initiation^8^ (Dagnew AF, unpublished).

With several promising vaccine candidates now advancing through phase 1 and 2 trials,^13^ the need for efficacy endpoints that allow for faster endpoint accrual is increasingly being recognised, together with trial designs that can better capture the full spectrum of bacteriologically confirmed tuberculosis disease.^3^^,^^4^^,^^9^^,^^14^

A potential concern is that tuberculosis vaccines, analogous to COVID-19 vaccines, for example, could confer greater protection against more severe clinical disease;^15^ more sensitive, symptom-agnostic screening strategies perceived to detect less severe disease or asymptomatic tuberculosis might, therefore, reduce measured vaccine efficacy, not because the vaccine is less effective against severe disease, but because the vaccine is being evaluated against a different disease spectrum. The clinical and public health significance of detecting and preventing this asymptomatic tuberculosis disease phenotype remains uncertain, as evidence regarding its contribution to morbidity, disease progression, or transmission is low.^9^^,^^16^, ^17^, ^18^, ^19^

The characteristics of asymptomatic bacteriologically confirmed tuberculosis are poorly understood. They may be highly similar to those of symptomatic bacteriologically confirmed tuberculosis, and therefore, may not represent a distinct disease state, but this remains uncertain. The clinical and public health relevance of asymptomatic bacteriologically confirmed tuberculosis might vary according to bacterial burden, persistence of microbiological positivity, radiographic evidence of pulmonary involvement, risk of progression to symptomatic disease, and potential contribution to transmission.^9^^,^^10^ These characteristics will become better known from an ongoing systematic review^20^ and empirical studies. This distinction is important for vaccine endpoint design because indiscriminate inclusion of transient or very low-burden bacteriological positivity could dilute measured vaccine efficacy and reduce clinical interpretability, whereas persistent or higher-burden asymptomatic disease could represent a biologically and public health-relevant vaccine-preventable state. Consistent with this uncertainty, WHO has highlighted asymptomatic tuberculosis as an emerging area for surveillance, treatment policy, and research, noting both that current tuberculosis treatment recommendations do not distinguish between symptomatic and asymptomatic tuberculosis and that further research is needed to identify optimal treatment approaches for asymptomatic tuberculosis.^10^

In this Health Policy paper, we outline how integrating symptom-agnostic screening and case verification could inform future tuberculosis vaccine efficacy trials, highlight key screening technology options measuring bacterial burden, estimate the potential effect of new strategies, and identify regulatory and operational considerations for their implementation. The search strategy for this narrative review is provided in the Supplementary Appendix.

## Orthogonal views of tuberculosis disease and the implications for endpoint design

In tuberculosis, neither diagnostic accuracy nor screening performance is an intrinsic or fixed property of a test, but a function of which dimension of disease is being measured and the population tested.^3^^,^^14^^,^^21^ Symptom-based screening captures the somewhat subjective experience of illness, microbiological assays reflect underlying mycobacterial burden, and imaging modalities index macroscopic pathology. Each approach, therefore, samples a distinct—yet overlapping in part—segment of the tuberculosis disease spectrum, and no single strategy can be assumed to represent disease in a uniform or comprehensive way.^14^ Framing screening strategies as orthogonal perspectives, rather than competing tools, provides a conceptual basis for understanding why different approaches yield divergent case counts, operational burdens, and the apparent vaccine effects.^14^^,^^22^, ^23^, ^24^

The diagnostic goal posts are moving with continued innovation. When Xpert MTB/RIF was introduced, for example, the benchmark for diagnostic accuracy shifted, as compared with that for smear microscopy. Xpert MTB/RIF, and later, the improved Xpert MTB/RIF Ultra, each detected a broader segment of the tuberculosis disease spectrum, including many smear-negative but culture-positive cases, thereby enabling early identification of a broader spectrum of individuals with lower or paucibacillary burdens and altering how the performance of other tests was interpreted.^25^, ^26^, ^27^

Building on this evolution, the latest diagnostic innovations are further expanding the detectable spectrum of tuberculosis of potential relevance for tuberculosis vaccine trials (table 1). Near point-of-care (NPOC) molecular technologies offer opportunities to identify bacteriologically confirmed disease earlier and with high specificity.^31^^,^^34^, ^35^, ^36^, ^37^ New imaging tools enable sensitive screening modalities and, when coupled with computer-aided detection software, reduce inter-reader variability bias.^34^^,^^38^^,^^39^ Spectrum-sensitive diagnostics, including tools reflecting bacterial burden (eg, in the form of semiquantitative categories or PCR cycle-threshold values) or the extent of pathology (eg, in the form of a computer-aided detection score) might add new dimensions to vaccine efficacy assessment. These innovations, when used to guide confirmatory testing and grade disease severity for trial endpoints, need to be analytically validated, clinically qualified for their intended use, and developed under quality and regulatory standards.^40^ As several new diagnostic tools begin to meet these requirements,^31^^,^^39^ expanding vaccine endpoints beyond symptom screen-positive disease could offer a more efficient and broader assessment of vaccine efficacy.^3^^,^^4^^,^^9^

**Table 1:** Emerging diagnostic and screening technologies for tuberculosis detection and their relevance to vaccine endpoint assessment

	Sample type	Key platforms or developers	Development stage	Performance (typical ranges)	Value for tuberculosis vaccine trials	Key limitations
LC-aNAATs	Sputum (provider-collected or self-collected); tongue swabs	Xpert MTB/RIF Ultra (Cepheid); Truenat MTB Plus/Ultima (Molbio)	Commercial; WHO-endorsed;^28^^,^^29^ widely implemented mWRDs	Sensitivity approximately 90% in symptomatic tuberculosis; lower in paucibacillary, asymptomatic, paediatric disease; specificity approximately 96–99%^29^	WHO comparator for bacteriological confirmation; strong regulatory acceptance; widely used in vaccine trials; semi-quantitative output reflects bacillary burden^16^^,^^30^	Reduced sensitivity in low-burden and asymptomatic disease; infrastructure and high costs; low feasibility for community-based sampling
Liquid culture (MGIT)	Sputum	BACTEC MGIT 960 (BD)	Commercial; WHO-endorsed	High sensitivity across the disease spectrum; specificity more than 99% (with species confirmation)^29^	Microbiological reference standard; detects viable bacilli; supports drug resistance testing; long history in vaccine trials	Slow (up to 42 days); requires Biosafety Level-3 lab, trained staff, reliable power; high cost; not field-deployable
NPOC molecular (tongue swab or sputum-dipped swab)	Tongue swab (provider-collected or self-collected); sputum-dipped swab	MTBC Nucleic Acid Test Card (Pluslife Mini Dock/Dock Pro 8); UniAmp *Mycobacterium tuberculosis* Nucleic Acid Test Card; other early-stage technologies	Pluslife commercially available, NPOC assay class endoresed by WHO-policy	Data from facility and community testing Tongue swab sensitivity approximately 75%, specificity approximately 99%; sputum-dipped swab sensitivity approximately 85%, specificity approximately 97%^31^^,^^32^	Non-invasive self-sampling; portable, battery-operated; rapid (<1 h); low cost; potential screening tool in community-based trial designs	Lower sensitivity than LC-aNAATs; low evidence base; reduced sensitivity in low-burden and asymptomatic disease^32^^,^^33^
Cell-free DNA and CRISPR-based assays	Blood, sputum, other fluids	IntelliGenome; TB-QUICK; Jiangsu MicroDiag; academic prototypes	Prototype or early translation	Early pilot studies, mostly laboratory-based, suggest good sensitivity in paucibacillary and extrapulmonary tuberculosis; specificity comparable to sputum molecular tests (small sample sizes)	Potential to detect low-burden and extrapulmonary tuberculosis; non-sputum sampling; can enable objective assessment of bacterial burden	Pre-commercial; low validation; operational feasibility unknown
Portable chest x-ray with computer-aided detection	Chest radiograph	Multiple hardware and computer-aided detection vendors	Commercial; WHO policy endorsed for screening	High sensitivity for symptomatic tuberculosis; lower specificity; reduced sensitivity for asymptomatic tuberculosis	Screening tool to triage for confirmatory testing; portable options; computer-aided detection reduces reader variability; possible use for end-of-study screening	Low specificity precludes confirmatory use; radiation restricts repeat use; integration into longitudinal trial designs evolving
Digital biomarkers (cough apps, wearables)	Audio signals; physiological data	Multiple developers; consumer devices	Early validation	Pilot cough app approximately 63% sensitivity, 88% specificity; broader evidence is limited	Non-invasive, scalable; continuous monitoring; potential adjunct for safety and efficacy signals; compatible with decentralised trial models	Immature evidence base; inadequate standardisation; not suitable for diagnostic endpoints

Summarised are the leading technologies under development or implementation for symptom-agnostic tuberculosis detection, including sample types, representative platforms, performance, advantages, and challenges in the context of tuberculosis vaccine trials. We distinguish between established reference standards (sputum-based molecular and culture-based assays) and novel tools under evaluation for NPOC and non-sputum-based detection. LC-aNAATs=low-complexity automated nucleic acid amplification tests. MGIT=Mycobacterial Growth Indicator Tube. mWRDs=molecular WHO-recommended rapid diagnostics. NPOC=near point-of-care. POC=point of care.

## Beyond symptoms: harnessing symptom-agnostic screening in future tuberculosis vaccine evaluation

Historical and contemporary data describe tuberculosis as a dynamic process, with individuals transitioning through phases of low and high disease activity over time.^41^^,^^42^ The split between symptomatic and asymptomatic tuberculosis is influenced by the sensitivity of the symptom screen, an individual’s willingness to report symptoms, and how symptoms are assessed.^19^^,^^43^ Asymptomatic tuberculosis often—but not uniformly—could represent earlier or less advanced disease and is frequently associated with lower mycobacterial burden, reflected in delayed time to culture positivity, lower smear grades, and lower semiquantitative or higher cycle-threshold values on molecular assays when sputum can be obtained.^11^^,^^19^^,^^35^^,^^42^^,^^44^, ^45^, ^46^ However, symptomatic tuberculosis also spans a broad biological spectrum, ranging from mild or intermittent symptoms with modest bacterial burden to advanced disease with extensive pathology and high bacillary load.^10^ Across both asymptomatic and symptomatic presentations, vaccine-preventable disease could be unlikely to align cleanly with symptom status alone. Instead, bacterial burden—closely linked to disease severity, progression risk, and transmission potential—might be more quantifiable and objective and could provide a more biologically coherent axis along which vaccine effects are expressed.^14^ This framing highlights that a central challenge is not whether asymptomatic or symptomatic tuberculosis should be detected, but whether the disease states captured as endpoints are biologically meaningful, vaccine-preventable, and interpretable within efficacy trials.

Screening participants irrespective of symptoms (symptom-agnostic screening) could offer a more inclusive strategy to trigger tuberculosis endpoint verification and address vaccine-preventable disease.^3^^,^^4^ The latest advances in molecular diagnostics now make this feasible: portable, highly specific, and affordable assays enable NPOC testing in communities, including self-sampling options as recommended by WHO.^31^^,^^36^ Diagnostic platforms with the ability to transmit results via Bluetooth, Wi-Fi or mobile networks, and emerging digital health tools allow for remote monitoring, data integration, and decentralised trial conduct.^47^^,^^48^

On one hand, more frequent testing during follow-up—whether through scheduled in-person visits, self-collected samples, or digitally triggered symptom checks—would detect tuberculosis earlier (figure). On the other, the high screening cadence in tuberculosis POD trials risks increasing the likelihood of transient, borderline results, or false-positive results, which can compromise endpoint specificity. Therefore, maintaining scientific, ethical, and regulatory rigour requires that tuberculosis vaccine trials continue to apply a stringent confirmation algorithm, typically demanding at least two independent positive bacteriological tests to define a confirmed case, and considering Xpert Ultra Trace calls as negative.^5^^,^^8^ As a result, broader screening triggers need not compromise endpoint specificity provided that confirmatory algorithms remain stringent and uniformly applied across trial arms.

**Figure:**
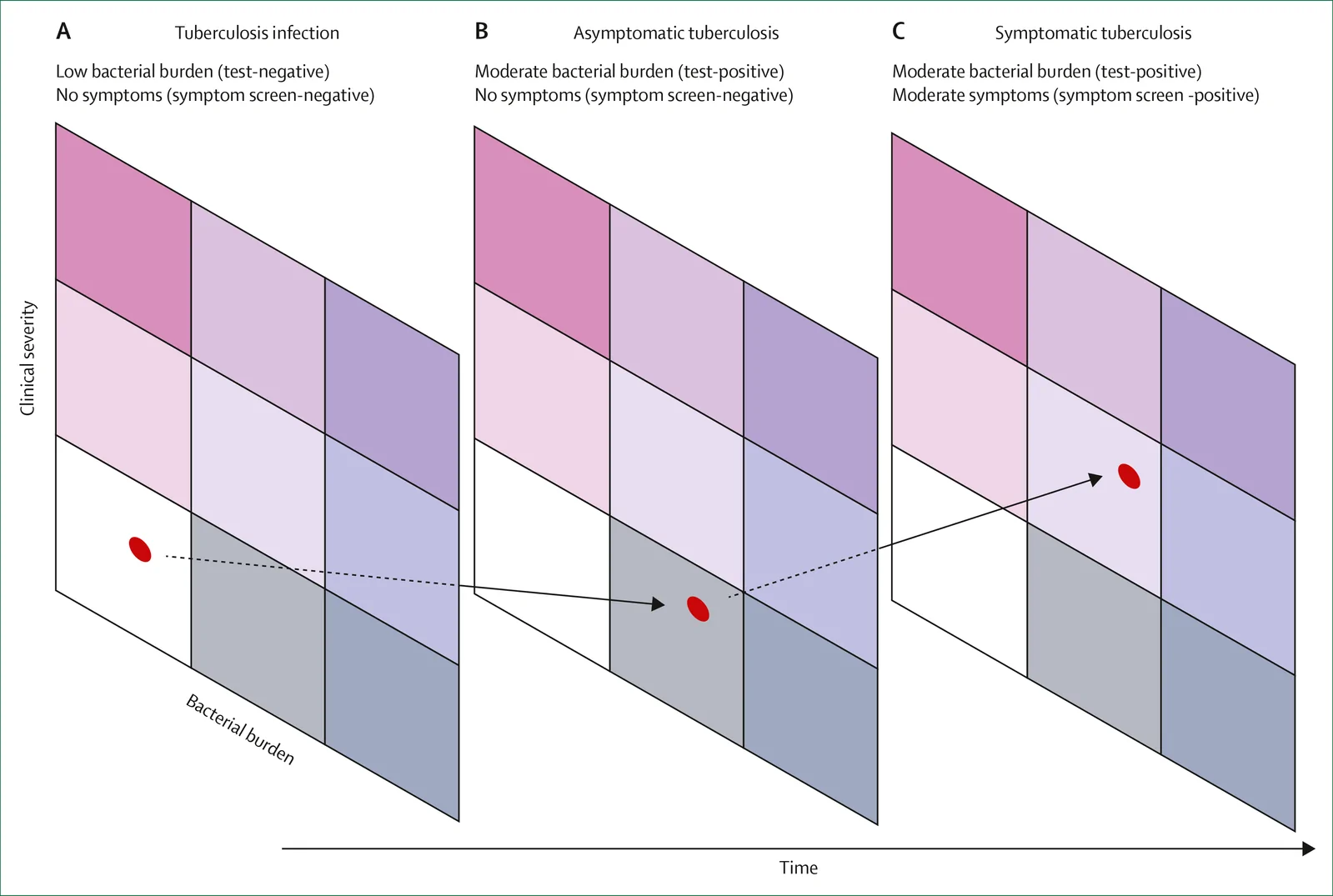
Tuberculosis disease dynamics, test positivity, and reporting of symptoms Conceptual illustration of evolution in the bacterial burden and experience of symptoms in a tuberculosis vaccine trial participant (red dot), from baseline (A) and through two rounds of symptoms screen and symptom-agnostic testing (B-C). In situation A (left), baseline, this individual is IGRA test-positive, has no symptoms, and due to the low bacterial burden, all microbiological tests are negative. In situation B (centre), asymptomatic tuberculosis, the bacterial load is sufficiently high to be detected with a test; however, the person screens symptom-negative and would not be flagged for confirmatory testing. In situation C, symptomatic tuberculosis, the person now has symptoms triggering a screening, the bacterial burden remains at a similar level as situation B, and the test would be positive at a case-verification visit. Note: Tuberculosis disease trajectories can be non-linear, fluctuating, or reversible; individuals can transition between asymptomatic and symptomatic states; and bacterial burden can fluctuate over time. Figure inspired by concepts introduced in the study by Kendall and colleagues.^14^ IGRA=interferon-γ release assay.

## Next-generation diagnostics: expanding the endpoint landscape

The diagnostic and screening technologies outlined in table 1 highlight complementary strengths for tuberculosis detection or screening triggers for confirmatory testing in tuberculosis vaccine trials. Sputum-based molecular WHO-recommended diagnostics, including low-complexity automated nucleic acid amplification tests, such as Xpert Ultra, and mycobacterial culture-based assays could already be used at regular intervals as symptom-agnostic screening tools in tuberculosis vaccine trials.^3^ However, logistical challenges associated with sputum collection, laboratory analysis, long time to result of culture-based assays, and the high cost, currently restrict their use in this capacity. Based on data from prior literature and preprints,^32^, ^33^, ^34^^,^^36^^,^^49^, ^50^, ^51^ among the next-generation tools addressing some of the limitations, NPOC tongue swab-based and sputum swab-based molecular assays are currently the most advanced new products, with late-stage platforms such as MTBC Nucleic Acid Test Card (Pluslife, China) and UniAmp (Huwel, India) showing, in facility and community settings, very high specificity, acceptable sensitivity (8–10% lower than that for Xpert Ultra), and strong usability features not met by the current sputum-based molecular diagnostics. Of note, WHO has now issued a strong recommendation for the use of NPOC molecular assays on tongue swabs and sputum in appropriate settings, further supporting their programmatic relevance and potential role in decentralised screening strategies.^31^ Based on data from prior literature the easier sampling associated with tongue-swab collection and high portability make them well suited for repeated, decentralised testing, potentially including self-sampling scenarios.^36^^,^^50^ Of note, the modest diagnostic sensitivity of NPOC, as compared with that of Xpert Ultra, suggests that this tool will detect primarily moderate and high bacterial burden tuberculosis disease, based on data from a preprint and prior literature,^31^^,^^32^^,^^36^^,^^51^ which the M72/AS01_E_ phase 2b trial suggested is vaccine preventable.^8^ Tongue-swab samples show stability at ambient temperature over several days,^46^ and NPOC instruments are low cost (eg, approximately US$335 for the platform at a $2·60 per test cost, as compared with the $19 000 for a standard GeneXpert IV, four-module instrument at a $7·97 per test cost^31^^,^^52^), aligning well with the WHO NPOC criteria defined in the target product profile updated in 2024.^53^ In contrast, cell-free DNA CRISPR-based diagnostics remain at an earlier stage of development but could offer unique value for detecting paucibacillary and extrapulmonary tuberculosis from both sputum-based and non-sputum-based samples such as blood in populations, aspects in which traditional sputum-based assays lead to challenges in access.^54^, ^55^, ^56^, ^57^, ^58^ Although promising, cell-free DNA-based assays require rigorous validation before they can be considered as primary endpoint tools in vaccine trials. As regulatory acceptance of these new tools evolves, they could ultimately extend endpoint detection into the earliest, presymptomatic stages of disease.^14^

Portable chest x-ray units paired with computer-aided detection software already carry WHO endorsement for tuberculosis screening of adults older than 15 years in communities and facilities and are valuable for their portability, high throughput, low per test cost, high sensitivity, and immediate interpretation.^11^^,^^12^^,^^39^^,^^59^ Although their specificity is lower than those of molecular assays, chest x-ray–computer-aided detection (CXR–CAD) can play an important complementary role as a sensitive screening tool or as a spectrum-sensitive measure of severity and pulmonary involvement.^60^ Digital tools such as cough apps, digital stethoscopes, POC ultrasound, and wearables are less mature but could offer scalable and passive surveillance capabilities, detecting subtle respiratory or physiological changes that can precede clinical disease.^42^, ^43^, ^44^ These approaches could also play a role maintaining participant engagement between scheduled visits.

Taken together, the innovations listed in table 1 illustrate a new and more diverse diagnostic and screening tool landscape. NPOC could provide the most practical near-term solution for microbiological rule-in testing; if mature products reach the market, then cell-free DNA assays may offer the highest sensitivity for early and extrapulmonary disease, CXR–CAD could provide the most robust option for radiographic monitoring, and digital health tools may contribute to the most scalable means for ongoing surveillance. Of note, strategic integration of these technologies not only promises a more comprehensive assessment of vaccine impact across the tuberculosis disease continuum but also have the potential to make trials faster and more cost-effective.^9^^,^^14^

## NPOC screening as a worked example of symptom-agnostic endpoint detection

To explore how symptom-agnostic screening might affect endpoint detection and case-verification burden, we developed a worked example using NPOC molecular testing. This example is intended to illustrate the implications of defined assumptions about screening sensitivity and specificity, rather than to provide a definitive estimate of overall trial efficiency. In this scenario, NPOC testing is not considered a stand-alone endpoint assay, but a decentralised screening trigger that would route screen-positive participants into the same type of confirmatory case-verification pathway used in the current trials.^5^^,^^6^

To provide an example of how screening test accuracy could influence key determinants of vaccine trial efficiency, we estimated the number of endpoints for an NPOC-based screening strategy, as compared with that for the M72/AS01_E_ phase 2b symptom screen, in a tuberculosis vaccine efficacy trial of 1663 participants followed-up for 36 months. Trial parameters were informed by the according-to-protocol analysis of the placebo arm of the M72/AS01_E_ phase 2b study,^8^ in which participants underwent a mean of 17·3 tuberculosis screening visits, yielding a total of 28 729 screening visits in the trial. Across these visits, 306 symptom screens were positive, of which 33 resulted in bacteriologically confirmed tuberculosis at case verification. Seven of these were initiated on tuberculosis treatment before presenting to the trial site for sputum sample collection and were excluded from the primary trial endpoint analysis (Dagnew AF, unpublished). A 2025 systematic review and meta-analysis of symptom screening with universal sputum microbiological testing found that only 52% of the bacteriologically confirmed tuberculosis cases were symptomatic.^11^ Under these assumptions, we estimated approximately 30·5 (48 asymptomatic/52 symptomatic × 33) additional asymptomatic tuberculosis cases in the trial population, yielding an estimate of 63 cases of bacteriologically confirmed tuberculosis in the control arm population of the M72/AS01_E_ phase 2b trial. Derived from these numbers, the sensitivity of the M72/AS01_E_ phase 2b symptom screen for detecting bacteriologically confirmed symptomatic tuberculosis (ie, the endpoint-capture process within the trial setting) was estimated to be 79% (26/33 × 100) and the specificity for bacteriologically confirmed tuberculosis 99% (1−[306−26]/[28 729−63]). NPOC test performance estimates were derived from the 2026 WHO evidence review of clinical evaluation studies involving symptomatic individuals presenting at facilities for care with signs and symptoms suggestive of tuberculosis and from community-based screenings.^31^^,^^32^^,^^36^ For the purpose of this analysis, we assumed the sensitivity for asymptomatic tuberculosis to be half of that observed in symptomatic tuberculosis, reflecting lower expected bacterial burden and supported by the community-based tongue-swab quantitative PCR data from a preprint and prior literature showing substantially lower sensitivity among household contacts and asymptomatic tuberculosis cases than among symptomatic clinic attendees (53% lower).^33^^,^^36^ Screening definitions and input parameters for sensitivity and specificity are summarised in table 2.

**Table 2:** Characteristics of symptom-based and symptom-agnostic screening strategies

	Definition	Sensitivity symptomatic tuberculosis	Sensitivity asymptomatic tuberculosis	Specificity
M72/AS01_E_ phase 2b symptom screen	One or more of the following symptoms: unexplained cough for more than 2 weeks, unexplained fever for more than 1 week, night sweats, unintentional weight loss, pleuritic chest pain, haemoptysis, shortness of breath on exertion, fatigue	79%	0%	99%
Symptom-agnostic NPOC	Near-patient molecular test of tongue swab sample	76%	38%	99%

Estimates for sensitivity and specificity are based on the placebo arm of the M72/AS01_E_ phase 2b trial, wherein 1663 participants underwent an average of 17·3 tuberculosis screening visits over 36 months (28 729 total). Among 306 positive symptom screens, 26 participants reported symptoms and had bacteriologically confirmed tuberculosis at screening visits; in addition, seven participants were initiated on treatment before sputum sampling (six met the third case definition, and one additional participant provided sputum more than 4 weeks after treatment initiation [Dagnew AF, unpublished]). Using meta-analysis data indicating that 52% of bacteriologically confirmed tuberculosis is symptomatic,^11^ we infer approximately 30 additional asymptomatic cases, for an estimated total of 63 cases. Under these assumptions, this analysis yields a sensitivity of 79% for symptomatic tuberculosis and specificity of 99% for symptom screening in detecting bacteriologically confirmed tuberculosis. NPOC accuracy estimates were from the WHO policy review of clinical evaluation studies mainly involving symptomatic individuals presenting at facilities for care with signs and symptoms suggestive of tuberculosis^31^ and sensitivity was reduced by half for asymptomatic tuberculosis given the anticipated lower bacterial burden in this population.^11^^,^^31^^,^^33^^,^^36^^,^^51^ NPOC=near point-of-care.

For symptom-agnostic NPOC screening, the numbers of screen-positive and bacteriologically confirmed tuberculosis cases detected were calculated (table 3).

**Table 3:** Yield of symptom-based and symptom-agnostic screening strategies

	Screen-positives, accrued	Bacteriologically confirmed detected, accrued
M72/AS01_E_ phase 2b, symptoms	306	26
Symptom-agnostic NPOC	267	37

Presented are the observed and expected number of screen-positive participants and number of tuberculosis cases detected during case-verification. Data from the M72/AE01_E_ phase 2b trial were derived from the study (Dagnew AF, unpublished). For M72 phase 2b, the numbers were observed in the study: for NPOC, we calculated accrued screen-positives as ([76% × 33] + [38% × 30] + [(1 – 99·2%) × 28 729] = 267); the accrued tuberculosis case yield was ([76·4% × 33] + [38·2% × 30) = 37). NPOC=near point-of-care.

The M72/AS01_E_ phase 2b symptom screen detected 26 symptomatic tuberculosis cases, whereas our worked example suggests the symptom-agnostic NPOC approach might accrue 37 tuberculosis endpoints, 1·4-times the yield of the symptom-based approach (table 3). This estimate should not be interpreted as a direct prediction of overall trial efficiency. In a randomised vaccine efficacy trial, symptom-agnostic screening could increase detection and confirmatory testing in both the placebo and vaccine arms and its net effect on trial duration, cost, statistical power, and measured vaccine efficacy would depend on the distribution of additional cases by trial arm, bacterial burden, symptom status, and confirmatory test positivity.

This worked example draws on observed screening yields from the M72/AS01_E_ phase 2b placebo arm and applies a simple, transparent framework to illustrate how screening test accuracy could influence endpoint accrual given the stated assumptions. For the purposes of this worked example, the seven participants who had initiated treatment outside the trial pathway were included only to approximate the total number of bacteriologically confirmed tuberculosis episodes observed in the placebo arm during follow-up. This finding should not be interpreted to mean that symptom-agnostic screening would necessarily have detected these participants before treatment initiation or prevented their exclusion from the primary endpoint analysis.

Symptom-agnostic screening with NPOC testing entails greater logistical demands than symptom screening conducted by telephone and in-person contact, including repeated tongue-swab collection, consumables, device availability, quality oversight, and participant time. With frequent screening events required in late-phase trials, these requirements need to be considered alongside the limitations of symptom-based approaches, which could delay or miss case detection and lead to treatment initiation outside scheduled trial visits. Careful assessment of operational and cost tradeoffs, also considering optimal screening cadence will, therefore, be essential when evaluating the feasibility and acceptability of symptom-agnostic screening strategies in tuberculosis vaccine trials. Protocols incorporating symptom-agnostic screening should also define procedures for referring participants who screen positive but do not meet trial endpoint criteria for appropriate clinical evaluation and subsequent management according to local standards of care.

Limitations of this worked example include reliance on a single trial context, inference of the true symptomatic and asymptomatic tuberculosis burden, NPOC sensitivity in asymptomatic disease from a diverse meta-analysis dataset, and extrapolation of NPOC performance from predominantly symptomatic, care-seeking populations.^31^ Although tongue-swab studies support lower sensitivity in asymptomatic and lower-burden disease,^32^^,^^33^^,^^36^^,^^46^ NPOC performance has not yet been assessed in interferon-γ release assay (IGRA)-positive, baseline Xpert-negative vaccine trial populations; the assumed sensitivity in asymptomatic disease should, therefore, be interpreted as an empirically informed scenario parameter rather than a validated estimate. Because the M72/AS01_E_ phase 2b trial enrolled IGRA-positive, symptom-negative, baseline single Xpert-negative participants, the symptomatic to asymptomatic prevalence ratio in non-trial populations may not directly apply to this trial population. Conversely, the key baseline concern is missed asymptomatic tuberculosis rather than tuberculosis infection itself, since IGRA positivity was an intended feature of the POD trial design.

The effect of symptom-agnostic screening on endpoint capture will also depend on access to non-trial tuberculosis services, the timing of scheduled screening visits, and the ability of trial systems to complete endpoint verification before treatment is initiated. Findings should be interpreted as indicative and warrant empirical evaluation in trial-like populations.

The worked example suggests that both the approaches had high specificity and low frequency of false-positive screens per visit, generating 306 case-verification visits for the symptom-based approach and 267 case-verification visits for the estimated symptom-agnostic approach, across the 17·3 screening rounds.

The low frequency of false-positive screens observed with the molecular NPOC assay is expected given the high analytical specificity of molecular testing. In contrast, the observed false-positive rate for symptom-based screening was lower than what was anticipated on the basis of the population prevalence surveys^22^ and household contact-tracing studies.^11^ This difference possibly reflects spectrum and selection effects inherent to the controlled trial setting, wherein participants were generally healthy, screened as symptom-negative at enrolment and confirmed Xpert MTB/RIF-negative, substantially reducing background symptom noise, as compared with that in the community-based screening.^22^ However, the seven TB cases initiated on treatment outside scheduled trial screening underscore the vulnerability of this approach to missing incident or asymptomatic disease earlier during follow-up.

Regular CXR–CAD screening using low-radiation dose portable chest x-ray systems offers an additional symptom-agnostic approach that could potentially be used in community-based trial settings. Although concerns regarding cumulative radiation exposure could preclude implementation at the same cadence as symptom-based or molecular screening, annual CXR–CAD during follow-up–or at the end of trial–cannot stand alone but could serve as a valuable complement to symptom-based screening by enabling detection of asymptomatic tuberculosis. A 2025 systematic review suggests that CXR–CAD will detect bacteriologically positive tuberculosis irrespective of symptoms with 62% sensitivity,^11^ at approximate 89% specificity.^22^ Using these numbers in our example from the M72/AS01_E_ phase 2b trial suggests 39 (62% × 63) tuberculosis cases detected and 459 (1663 × (1–89%) × 3) screen-positives accrued with annual screening for 3 years.

The estimates presented herein are more conservative than previous estimates from dynamic modelling, which suggested up to 2·2-times as many endpoints with a symptom-agnostic approach using the more sensitive gold-standard sputum liquid mycobacterial culture (Mycobacterial Growth Indicator Tube [MGIT]) and Xpert Ultra at all screening visits (design 4).^3^ Although the logistics and cost of sputum collection, parallel culture, and Xpert Ultra on all trial participants at every screening visit and 42-day incubation required for a culture-based test to be deemed negative might render this approach challenging to use;^61^ it highlights opportunities in the approach to symptom-agnostic screening and emphasises the need for empirical data to understand the effect of symptom-agnostic screening on the ratio of symptomatic to asymptomatic tuberculosis detected during follow-up and bacterial loads among those individuals.

## Aligning baseline screening, follow-up cadence, and endpoint interpretation

The interpretation of endpoints detected by symptom-agnostic screening depends on the alignment between baseline screening, follow-up screening cadence, and endpoint definition. The sensitivity of the assessments at enrolment determines which types of tuberculosis are excluded at baseline and, consequently, influences in part the types of tuberculosis captured as incident endpoints during follow-up. In the current M72/AS01_E_ and MTBVAC POD trials for adults and adolescents, enrolment requires participants to be IGRA-positive, symptom screen-negative, and negative by one Xpert MTB/RIF or Xpert MTB/RIF Ultra test, thereby excluding most prevalent microbiologically detectable disease while retaining individuals at elevated risk of progression.^5^^,^^6^ The screening approach contributes to ascertaining both endpoint composition and vaccine efficacy estimates, underscoring the need to align enrolment criteria, screening cadence, and endpoint definitions to ensure interpretability and relevance.

As discussed earlier, broader, and more sensitive screening could shift detected endpoints towards milder or earlier disease states, with potential implications for measured vaccine efficacy. Apparent reductions in efficacy under such approaches could, therefore, reflect differences in the disease spectrum captured rather than diminished biological protection generated by the vaccine (type II error). An open question remains whether less frequent screening cadence with sensitive molecular tools can achieve an optimal balance between capturing microbiologically confirmable incident tuberculosis, avoiding preferential detection of low-burden disease that could be less vaccine preventable, and minimising case detection outside the trial.

In the M72/AS01_E_ phase 2b trial placebo group, only three of the 26 accrued endpoints occurred within the first 9 months, suggesting that baseline screening effectively excluded most prevalent asymptomatic disease.^8^ Vaccine efficacy was 50% against symptomatic tuberculosis defined by at least one of six bacteriological tests being positive (13 cases in the M72 group *vs* 26 in the placebo), increasing to 68% in the prespecified sensitivity analysis requiring confirmation by at least two of six bacteriological tests (7 *vs* 22), and in the second case definition requiring detection by Xpert MTB/RIF (8 *vs* 21) and a vaccine efficacy of 62%. The increasing disparity in case counts between placebo and vaccine groups with confirmation based on a greater number of positive tests per case, or detection by the less sensitive Xpert MTB/RIF assay, is consistent with both a reduction in false-positive results and higher bacterial burden among placebo recipients than in vaccinated participants, suggesting stronger vaccine protection against tuberculosis characterised by higher bacterial burden.^8^^,^^62^^,^^63^

These considerations might caution against replacing symptom-based screening solely to avoid oversampling asymptomatic and presumed early or milder stage-tuberculosis, or both, which might not be vaccine preventable to the same degree. However, empirical evidence is needed to better understand bacterial burden among individuals with asymptomatic and symptomatic tuberculosis in a trial population. Instead, symptom-agnostic triggers that are directly coupled to bacterial burden might preferentially capture the high bacterial burden and vaccine-preventable disease and, thereby, increase measured vaccine efficacy without the bias of subjective symptom reporting. Conceptually, symptom-based and burden-linked symptom-agnostic screening represents orthogonal views of tuberculosis disease—one grounded in more subjective experience and the other in underlying mycobacterial burden—rather than competing approaches to endpoint ascertainment (figure).

This distinction is particularly relevant for NPOC, wherein the symptom-agnostic screening example described previously illustrated the higher endpoint yield across symptomatic and asymptomatic tuberculosis cases, whereas, at the same time, the lower analytical sensitivity as compared with those of MGIT and Xpert Ultra inherently de-emphasised low-burden disease, such as trace, very low, or low semiquantitative results^35^^,^^46^—a part of the disease spectrum that could be identified in high-cadence symptom screening, yet less likely to be vaccine preventable.

Assuming that tuberculosis typically progresses towards higher bacillary burden over time, a lower-cadence symptom-agnostic NPOC screening schedule would be expected to identify a greater proportion of screen-positive cases at higher bacterial burden, increasing the likelihood of confirmatory test positivity and reducing the risk of screening out low-burden endpoints through early treatment. Empirical data will be needed to define the optimal cadence, balancing endpoint yield and microbiologic confirmation, participant safety and timely care, and real-world behaviour, including the likelihood that participants seek care outside the trial between scheduled screens, which could dilute differences between screening strategies.

## Navigating regulatory pathways for novel endpoints and trial designs

Definitions of disease in tuberculosis vaccine efficacy trials are evolving with advances in diagnostic technologies, as illustrated by the M72/AS01_E_ programme—its phase 2b trial used a Xpert MTB/RIF and MGIT culture for symptom-triggered confirmation,^8^ whereas the ongoing phase 3 trial has adopted the more sensitive Xpert Ultra^64^ alongside MGIT culture^5^ to align with the updated WHO diagnostic recommendations. Similarly, when WHO updated its guidelines on symptom-based tuberculosis screening in 2021,^22^ the ongoing M72/AS01_E_ phase 3 trial moved to a less stringent definition of symptoms.^5^ Both the MTBVAC phase 2b and M72/AS01_E_ phase 3 trials required three sputum samples and two diagnostic tests per sputum sample per presumptive case. However, although the M72/AS01_E_ phase 2b trial used a primary endpoint defined by at least one of six sputum tests being positive, the phase 3 trial adopted a primary endpoint requiring confirmation by at least two positive sputum tests of six to reduce false-positive detection.^5^

This distinction between an established endpoint procedure and a new microbiological measure has precedent in tuberculosis drug development. Time to positivity in automated liquid culture was introduced as an alternative to manual colony-forming unit counts in early bactericidal activity studies only after studies showed that the measure correlated with established quantitative culture methods and could be used for a defined purpose.^65^ The relevance for tuberculosis vaccine trials is that NPOC or other symptom-agnostic tools should not be treated as replacements for endpoint detection simply because they are operationally attractive or WHO-recommended. Instead, they should first be evaluated alongside established case-verification procedures, with their role defined as screening triggers, exploratory measures, or potential endpoint components.^66^

Although treatment guidelines recommend therapy for all test-positive individuals at risk, regardless of symptoms,^10^^,^^22^ vaccine trials have traditionally relied on symptom-triggered case definitions.^4^ For vaccines, regulatory acceptance of broader, symptom-agnostic endpoints will require evidence that preventing asymptomatic tuberculosis translates into measurable reductions in morbidity, mortality, and transmission.^9^^,^^67^^,^^68^ Establishing this linkage represents an important scientific and policy frontier, particularly as new diagnostics now allow for detection of new disease states and populations at risk with high specificity.^3^^,^^9^^,^^14^^,^^16^^,^^30^

To navigate this transition effectively, early and structured engagement with regulatory authorities and other stakeholders, including the WHO Product Development for Vaccines Advisory Committee, WHO Strategic Advisory Group of Experts on Immunization, and WHO Technical Advisory Group on Evidence for Clinical and Policy Considerations for New Tuberculosis Vaccines, in addition to regional and national immunisation technical advisory groups, will be essential. Such dialogue can help to inform recommendations and decisions about acceptability and policy translation related to symptom-agnostic and spectrum-sensitive endpoints.^69^

Experience from other vaccine fields illustrates that endpoint selection depends on disease biology, public health relevance, feasibility, and regulatory acceptability. In COVID-19 vaccine trials, asymptomatic infection was sometimes assessed through active surveillance such as repeated swabbing or serology, although pivotal efficacy analyses generally prioritised symptomatic or clinically more severe disease, because these endpoints were more directly interpretable and operationally feasible.^70^, ^71^, ^72^ HPV vaccine development provides a complementary example: earlier biological endpoints, such as persistent HPV infection or high-grade cervical lesions, became acceptable only after strong evidence linked them to later cancer risk and after regulatory and policy alignment.^73^^,^^74^ These examples reinforce that symptom-agnostic screening tools as contributors to case ascertainment in tuberculosis vaccine trials will require careful qualification before they can be used in pivotal efficacy assessment.^9^

Distinguishing programmatic diagnostic policy from regulatory acceptability will also be important for definition and regulatory acceptance of licensure-trial endpoints. The WHO recommendation of an NPOC diagnostic class supports public health relevance and implementation feasibility but does not by itself establish that a specific assay, specimen type, or decentralised testing procedure will be acceptable to stringent regulatory authorities for use in vaccine efficacy endpoints. Such use will require intended use-specific analytical validation, clinical qualification in trial-like populations, standardised specimen collection and quality systems, and early agreement with regulators on whether the tool is used as a screening trigger, to define an exploratory endpoint, secondary endpoint, or as a component of a licensure trial endpoint definition.

With WHO’s strong policy recommendation for the NPOC class using sputum and tongue-swab samples, including self-collected specimens, these assays are now positioned for broader programmatic use in appropriate settings.^31^ This policy shift might increase familiarity, operational experience and confidence in these tools, which could in turn facilitate their consideration for bacteriological confirmation of endpoints in tuberculosis vaccine trials—particularly where decentralised testing is advantageous—while potentially strengthening their regulatory acceptability.^31^

## A staged pathway for evaluating symptom-agnostic strategies to define vaccine efficacy trial endpoints

Symptom-agnostic screening, enabled by validated and policy-endorsed next-generation diagnostics, could expand how tuberculosis vaccine efficacy is measured, although these approaches should be evaluated through a staged evidence-generation pathway rather than adopted immediately as a replacement for current symptom-triggered endpoints. In the near term, symptom-agnostic screening could be incorporated into longitudinal cohort studies or nested studies within vaccine trials, wherein specimens are collected but not analysed until the end of the trial. Such studies allow characterisation of the frequency, bacterial burden, symptom status, radiographic features, persistence, and clinical outcome of bacteriologically positive disease detected outside symptom-triggered pathways.

A next step would be to evaluate these approaches as exploratory or secondary endpoint-capture pathways alongside established POD endpoint-capture pathways. Such studies should quantify diagnostic performance in the intended-use setting and population, the number of screen-positive participants requiring case verification, the proportion meeting stringent bacteriological endpoint definitions, and the effect of alternative screening strategies on vaccine efficacy estimates, case-verification burden, cost, and trial operations.

Only after analytical validation, clinical qualification, and prospective evidence linking bacteriologically confirmed disease detected by symptom-agnostic screening to clinical outcomes should endpoints defined by these methods be considered for use as co-primary or primary endpoints in pivotal tuberculosis vaccine trials.

These steps should be undertaken with strict adherence to quality and data standards, to ensure that novel endpoints are scientifically sound, interpretable, and policy relevant. If implemented proactively, then these approaches could help to define whether endpoints defined by symptom-agnostic approaches can support more efficient and interpretable tuberculosis vaccine trials, potentially shortening the path from vaccine candidate to measurable public health impact, helping to deliver effective tuberculosis vaccines to those who need them the most, sooner.

## Declaration of interests

MH declares research grants from the University of Cape Town. GC declares research grants from The Aurum Institute. AFD is an employee of the Gates Medical Research Institute, which is involved in tuberculosis vaccine development. AP-N was hired as a paid consultant for Molbio Diagnostics post-submission (March 2026). Molbio Diagnostics manufactures diagnostics products for tuberculosis listed in this manuscript. The authors hold no patent or claim to commercial products associated with research to this product. The other authors declare no competing interests.
