# Supplementary material for: Symptom-agnostic endpoints in tuberculosis vaccine efficacy trials
Source: Lancet Glob Health. 2026 Oct 1;14(11):None. doi: 10.1016/j.langlo.2026.104079 (PMC13755382; doi:10.1016/j.langlo.2026.104079)
Supplement: Supplementary file 1 [file mmc1.pdf]

THE LANCET
Global Health

Supplementary appendix

This appendix formed part of the original submission and has been peer reviewed. We post it as supplied by the authors.

Supplement to: Ruhwald M, Dagnew AF, Churchyard G, et al. Symptom-agnostic endpoints in tuberculosis vaccine efficacy trials. *Lancet Glob Health* 2026. https://doi.org/10.1016/j.langlo.2026.104079

Search strategy and selection criteria

This narrative review was informed by targeted searches of PubMed, ClinicalTrials.gov, WHO publications and policy documents, trial protocols, relevant preprint servers, and the reference lists of identified articles. Searches were conducted for English-language material published from Jan 1, 2000, to July 15, 2026, with earlier seminal publications included wherever relevant. Search terms were used alone and in combination and included “tuberculosis”, “TB vaccine”, “vaccine efficacy trial”, “prevention of disease”, “endpoint”, “case definition”, “asymptomatic tuberculosis”, “subclinical tuberculosis”, “symptom screening”, “symptom-agnostic screening”, “bacterial burden”, “near point-of-care”, “molecular diagnostic”, “tongue swab”, “Xpert”, “culture”, “chest radiography”, “computer-aided detection”, “CAD”, “cell-free DNA”, “CRISPR”, and “digital biomarkers”. We prioritised studies, trial protocols, systematic reviews, diagnostic accuracy studies, and WHO or regulatory guidance directly relevant to tuberculosis vaccine endpoints, screening technologies, and bacteriological confirmation. Studies were excluded when they did not address tuberculosis detection, vaccine-trial endpoint ascertainment, or the intended use of the technologies discussed. Selection was based on relevance rather than on formal quality scoring, and the search was not intended to be systematic or exhaustive.
